# Hyperplastic Polyps Are Innocuous Lesions in Hereditary Nonpolyposis Colorectal Cancers 

**DOI:** 10.1155/2011/653163

**Published:** 2011-06-07

**Authors:** D. Speake, J. O'Sullivan, D. G. Evans, F. Lalloo, J. Hill, R. F. T. McMahon

**Affiliations:** ^1^Colorectal Unit, Department of Surgery, Manchester Royal Infirmary, Manchester M13 9WL, UK; ^2^North West Genetics Reference Laboratory, St. Mary's Hospital, Manchester M13 9WL, UK; ^3^Department of Clinical Genetics, St. Mary's Hospital, Manchester M13 9WL, UK; ^4^Department Histopathology, Manchester Royal Infirmary, Manchester M13 9WL, UK; ^5^Department of Laboratory and Regenerative Medicine, The University of Manchester, Manchester M13 9PL, UK

## Abstract

*Aims*. To compare methylation profiles, protein expression, and microsatellite instability (MSI) of sporadic, HNPCC, and familial hyperplastic polyps (HPs). *Methods*. Methylation-specific PCR (MSP) and pyrosequencing assessed p16, MGMT, hMLH-1, MINT 1, and MINT 31 methylation. IHC (Immunohistochemistry) assessed Ki67, CK20, hMLH-1, hMSH-2, and hMSH-6 protein expression. MSI analysis was performed on those polyps with adequate DNA remaining. *Results*. 124 HPs were identified 78 sporadic, 21 HNPCC, 25 familial, and the HNPCC group demonstrated no significant differences in overall methylation (*P* = .186 Chi^2^). The familial group demonstrated significantly less over all methylation levels (*P* = .004 Chi^2^). *Conclusions*. HPs that occur in HNPCC have no more worrying features at a molecular level than those patients with HPs in a sporadic setting.

## 1. Introduction

For many years hyperplastic polyps (HPs) have been considered innocuous lesions. Recent pathological and molecular observations have challenged this and given rise to the serrated adenoma carcinoma sequence. Prior to this, the adenoma carcinoma sequence was believed to be the mechanism by which most or all colorectal cancer (CRC) occurred; this sequence described a series of mutations in genes resulting in an increasingly dysplastic adenoma progressing to CRC over time. However, the reported accumulation of genetic changes described in the adenoma-carcinoma sequence [1] does not wholly account for neoplastic transformation within the colon [2–4]. 

Mutation of the mismatch repair genes (most commonly MLH-1 and MSH-2) leading to MSI (microsatellite instability) in CRC is an alternative mechanism underlying tumour formation in patients with HNPCC [5]. It is now known that up to 15% of sporadic CRC also have MSI; not as a consequence of mutation in the mismatch repair genes but through a process of epigenetic changes to MLH-1 [6].

Epigenetics describes the silencing of key tumour suppressor genes through methylation of cytosine residues in the promoter regions of DNA [7]. Methylation occurs in up to 30% of CRC [8]. Hyperplastic polyps and serrated adenomas (SAs) have also been demonstrated to develop as a consequence of methylation. That methylation is present in HPs, SAs, and 30% of CRC without the series of mutations described in the adenoma carcinoma sequence has led to the suggestion of a serrated neoplasia pathway [7, 8]. 

A number of known genes are silenced in cancer by methylation including MLH-1, p16^INK4a^, MGMT, and MINT 1 and 31. Methylation affects gene expression in a graded fashion, a threshold of 15% promoter methylation is considered biologically significant [9–11]. The number of genes methylated can be described by CiMP (CpG island methylated phenotype): CiMP S, no methylation; CiMP L, one gene methylated, and CiMP H, more than one gene methylated [11, 12]. 

Most studies of sporadic HPs are either on patients with hyperplastic polyposis or selected HPs based on location in the colon or size. It is known that methylation is a frequent event in sporadic HPs [13, 14]; however, the true incidence of methylation in HPs from a sporadic *unselected* cohort remains unknown. In the HNPCC screening program at Manchester Royal Infirmary 49% of polyps detected were HPs or SAs [15]. There are few papers describing the incidence of HPs in HNPCC and incomplete data regarding the molecular profiles of these lesions [16–18]. Mismatch repair mutations are considered the pathway by which cancer develops in patients with HNPCC. There is no evidence to date that those HPs that occur in HNPCC arise as a consequence of germline mismatch repair mutation and therefore should arise as a consequence of methylation like there sporadic counterparts. The methylation profile of HPs and SAs in patients with a family history of CRC is unknown. This study analyses methylation profiles of HPs which arise in patients with a greater than 1 : 10 empiric risk of CRC (familial group), patients with HNPCC, and patients who develop HPs sporadically. In addition Ki-67 and CK-20 immunostaining has been performed to analyse mucosal proliferation and differentiation as previously described [19–21].

## 2. Methods

### 2.1. Subjects and Recruitment

**Figure 1:**
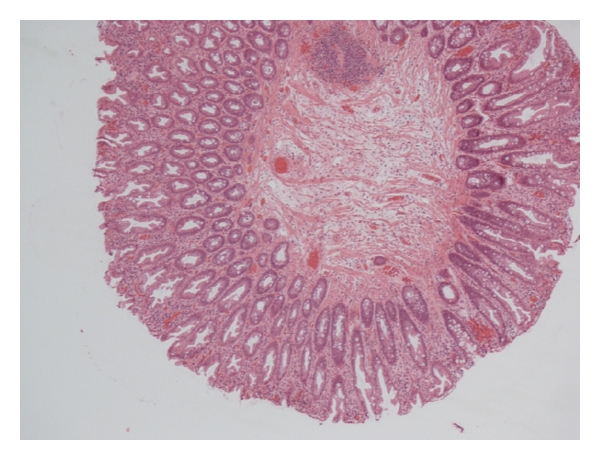
Low-powered view of a typical HP processed for the study.

Patients were recruited with informed consent via the Manchester Royal Infirmary Endoscopy Unit (sporadic) and the regional genetics service HNPCC database (familial and HNPCC). Sporadic hyperplastic polyps were defined as polyps occurring in individuals without a family history of colorectal cancer; these contained an unselected 12-month cohort of specimens. Familial polyps were defined as polyps occurring in individuals with a greater than 1 : 10 empiric risk of CRC; their risk of was obtained from genetic records and scrutiny of pedigrees. Patients with HNPCC were identified via the regional genetics service. Ethical approval was obtained from South Manchester LREC 05/Q1403/109. All slides were reviewed by a consultant histopathologist to confirm presence of HPs in the tissue blocks (Figure 1).

### 2.2. Methylation Analysis

DNA extraction from paraffin embedded tissue was performed with a DNeasy kit (Qiagen Crawley, UK). Bisulfite modification of polyp DNA was performed using an EZ DNA methylation kit (Zymo Research, California, USA). Methylation analysis was performed with a combination of methylation specific PCR (MSP) for p16^INK4a^ and MGMT, and MLH-1 and pyrosequencing for MINT 1 and MINT 31. Both methods have been previously well described [22–25]. Negative controls were generated from leukocyte DNA and positive controls from the SW48 cell line. The primers and PCR conditions for MSP are described in Tables 1–4. Greater than 15% methylation was considered biologically significant when quantifiable techniques were employed.

**Figure 2:**
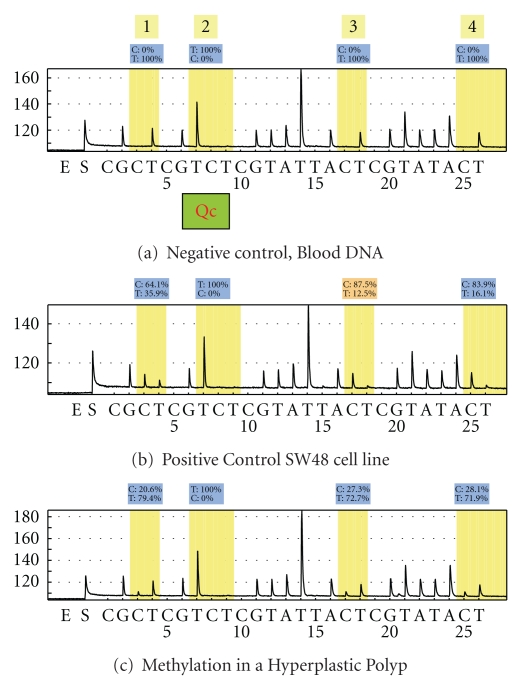
Pyrograms for MINT 1. (a) Represents unmethylated control bisulfite modified DNA. Position 2 represents the quality control loci and should remain negative for Cs. It can be seen that all Cs within the CpG islands at positions 1, 3, and 4 also reveal no Cs, that is, the DNA is unmethylated. In contrast, pyrogram B which represents a positive control from the SW48 cell line, position 2 remains negative for Cs, but the CpG's at position 1, 3, and 4 have 64.1%, 87.5%, and 83.9% methylation, respectively. This positive control as anticipated is methylated. The final sample in C represents DNA extracted from a hyperplastic polyp. This demonstrates 20.6%, 27.3%, and 28.1% methylation at position 1, 3, and 4, respectively, with a negative result for the internal control at position 2.

For methylation analysis of MINT 1 and MINT 31, primers were designed for the designated sequence as described by Toyota using pyrosequencing software provided by biotage (www.biotagebio.com), Table 5 and Figure 2 [11, 25]. The accuracy of pyrosequencing was confirmed by generating standard curves as previously described [25]. Pyrosequencing generated quantifiable results for methylation, a 15% threshold for methylation was considered biologically significant. Results for both MSP and pyrosequencing were recorded in an SPSS and Excel spreadsheet for statistical analysis.

### 2.3. Immunohistochemistry

Immunohistochemistry was performed on paraffin embedded tissues from 5 *μ*M sections cut on a microtome and mounted on Surgipath positively charged slides (Peterborough, UK). Automated IHC was optimised using the TECHMATE 500 (DAKO, UK) and EnVision (DAKO, UK) detection system. Steam antigen retrieval was performed for 3 minutes in TRIS EDTA pH 9.0 for MLH-1 and MSH-6 and 0.01 mM EDTA for MSH-2, PMS-2, Ki 67, and CK 20. Antibodies were purchased from BD pharmingen, concentrations were as follows: MLH-1 1 : 75, MSH-2 1 : 200, MSH-6 1 : 20, Ki-67 1 : 200, and CK 20 1 : 200. 

In total 167 hyperplastic polyps were analysed: 115 sporadic and 52 familial. A substantially larger number of sporadic hyperplastic polyp were analysed in comparison to methylation analysis since many of the smaller polyps either failed DNA extraction, were too small to consider for DNA extraction, or did not have adequate tissue in the block to perform DNA extraction. All polyps tested for methylation had IHC analysis with all 5 antibodies.

### 2.4. MSI

A panel of 5 markers: BAT25, BAT 26, NR-21, NR-24, and MONO-27 were used. These are all mono-nucleotide markers which are considered more sensitive for MSI than the original panel of markers recommended by the American Joint Commission on Cancer [26, 27]. Microsatellite analysis was performed using previously described techniques [26, 27].

### 2.5. Statistics

Comparison between the sporadic and familial groups were made using the Fisher's exact test where numbers were less than 5 or Chi^2^ where numbers exceeded 5. SpSS and Excel software was used to perform statistical analysis. Correction for multiple comparisons were made.

## 3. Results

After tissue processing for methylation analysis there were 124 hyperplastic polyps with adequate amounts of DNA. There were 78 sporadic versus 25 familial polyps and 21 HNPCC polyps. Of the 124 polyps, 17 were from the right side of the colon (proximal to the splenic flexure) and 96 from the left side of the colon. For 11 polyps there were no records documenting position. The male-to-female ratio was 1 : 1, aged 22–78 years (median 47). 

The overall incidence of methylation in all hyperplastic polyps tested were MLH-1 10%, p16^INK4a^ 26%, MGMT 19%, MINT 1 28%, and MINT 31 26%. When CiMP was calculated for the 124 hyperplastic polyps 36% (*n* = 45) were CiMP stable, 34% (*n* = 42) were CiMP low, and 30% (*n* = 37) were CiMP high. This results in an overall CiMP + rate of 64%. 

**Figure 3:**
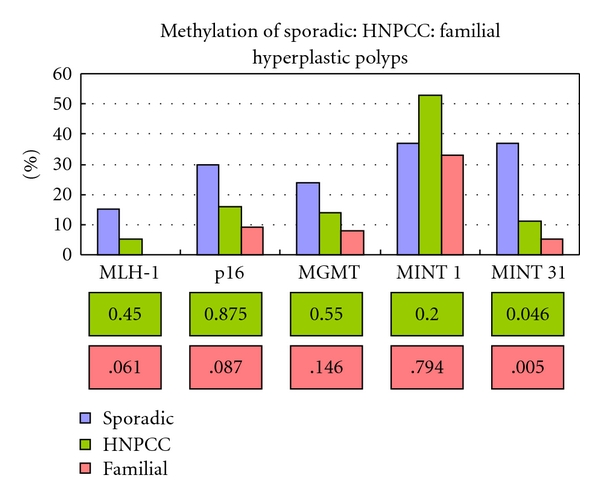
Methylation of hyperplastic polyps, sporadic: HNPCC: familial.* P* values are in pink boxes. No significant differences were demonstrated between the sporadic and HNPCC groups. For MINT 31 there was a significant difference between the familial and sporadic group. For MLH-1 (MSP), p16^INK4a^ and MGMT the difference between the familial and sporadic group nearly reached significance.

**Figure 4:**
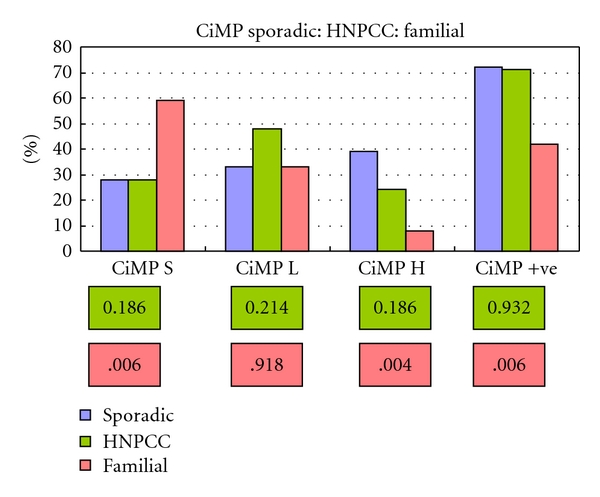
CiMP for sporadic: HNPCC: familial hyperplastic polyps. *P* values (Chi^2^) in pink boxes. No significant difference were observed between the sporadic and HNPCC group (olive boxes). Comparing the pattern of methylation after scoring CiMP for the sporadic and familial groups demonstrated a significant difference for CiMP H and overall CiMP positive status (pink boxes).

For the HNPCC group there were no significant differences in the incidence of methylation in comparison to the sporadic group except for MINT 31 *P* = .046 (Fisher's exact test). These observations were further confirmed when comparing CiMP status where no significant differences were seen between CiMP low (*P* = .214), CiMP high (*P* = .186), and CiMP  + (*P* = .932) status (Figures 3 and 4).

The familial group demonstrated a significantly lower incidence of methylation of MINT 31 when compared to the sporadic cohort and nearly reached significance for MLH-1, p16^INK4a^, and MGMT. When CiMP status was plotted, significant differences were observed between CiMP stable, high, and overall CiMP +ve rates. CiMP low did not demonstrate a significant difference between either the HNPCC or familial group (Figures 3 and 4).

**Figure 5:**
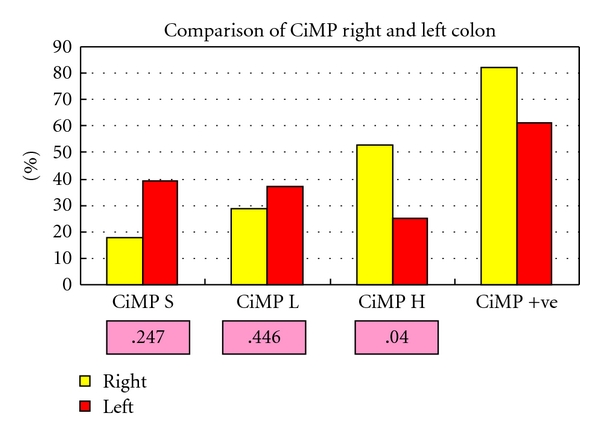
CiMP for left- and right-sided HPs, *P* values (Chi^2^) pink boxes.

In total, 17 hyperplastic polyps were right sided. methylation was more frequent in the right side of the colon for p16^INK4a^, MINT 1, and MINT 31 (*P* = .019,  .010 and  .012, resp., Chi^2^) but not for MGMT (*P* = .518 Fisher's exact test) or MLH-1 (*P* = .199 Fisher's exact test). Although over double the amount of polyps from the right side of the colon had MLH-1 methylation detected by MSP (9% left versus 19% right) this difference did not reach significance (*P* = .199 Fisher's exact test). However, when CiMP was compared between the left and right sides of the colon (Figure 5) CiMP high was more frequently encountered in HPs from the right side of the colon (*P* = .040  Chi^2^).

### 3.1. MSI Hyperplastic Polyps

After methylation analysis there was enough DNA remaining to perform MSI analysis on 81 hyperplastic polyps. MSI-H was not detected in any of the hyperplastic polyps, MSI-L was detected in 3 (5.5%) of the sporadic and 1 (3.7%) of the HNPCC hyperplastic polyps, these differences were not significant (Fisher's exact test 1.00). MLH-1 methylation was found in all four specimens demonstrating MSI-L.

### 3.2. Details of Immunohistochemical Analysis of Hyperplastic Polyps

**Figure 6:**
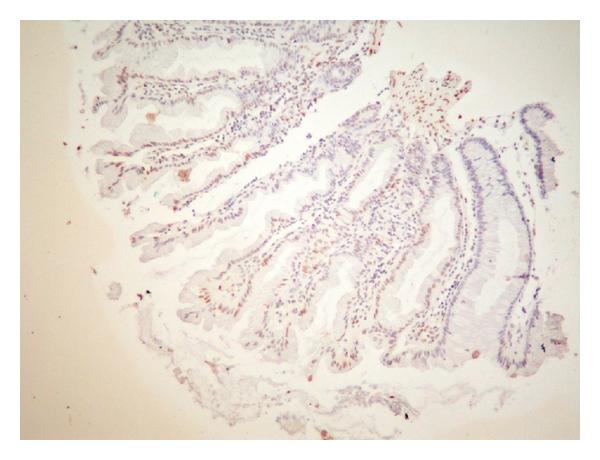
High-powered view of HP showing weak staining with MLH-1 protein and known MLH-1 methylation.

**Figure 7:**
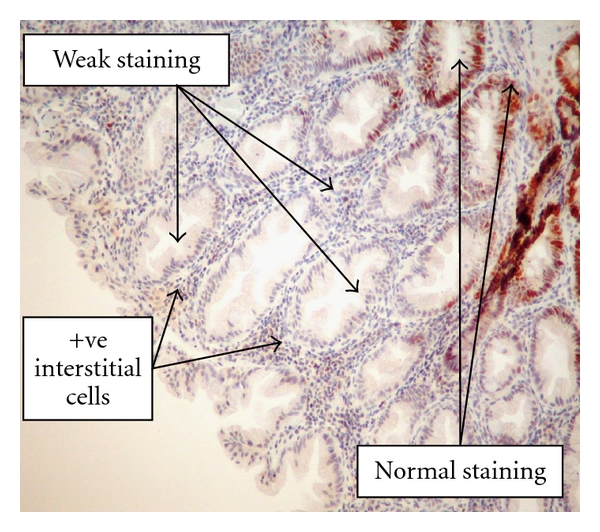
High-powered view demonstrating weak MSH-6 methylation in a hyperplastic polyp.

The results for IHC of the mismatch repair protein MLH-1, MSH-2, and MSH-6 can be seen in Table 6. None of the polyps demonstrated complete loss of staining. Weak staining was encountered with all three antibodies. This was believed to be related to poor tissue fixation in the majority of cases. However, those polyps which were methylated at the MLH-1 promoter region frequently showed weak staining with the MLH-1 antibody (10%; Figure 6). MSH-6 frequently showed weak staining in 47 (28%) of the hyperplastic polyps analysed (Figure 7).

The normal pattern of expression for Ki67 is in the base of the crypts and for CK20 the luminal surface of the crypts. Staining for Ki67 was predominantly basal or to the middle third of the crypts, more extensive staining to the outer third of the crypts was seen in 12/167 cases. Staining for CK20 was predominantly localised to the outer and middle third, although more extensive staining into the base of the crypts was seen in over one-third of cases Table 7.

## 4. Discussion

Analysis of methylation profiles of HPs from patients with a sporadic and familial risk of CRC has not previously been performed. This study has shown that in an HNPCC screening program, HPs that occur in those patients with confirmed mutations or HNPCC on clinical grounds have no more worrying features at a molecular level than those patients with HPs in a sporadic setting. Thus, those HPs that occur in patients with HNPCC could possibly be managed in the same manner as those that are detected in other clinical scenarios. 

The safety of leaving HPs in HNPCC patients should be further confirmed as those in a screening program will receive alternate year colonoscopy. This new data may offer a novel contribution to the management of HNPCC patients. If so, patients with HNPCC who have HPs removed can be reassured these lesions are not manifestations of their condition. To validate these results a retrospective power study was performed looking for a 10% difference between the sporadic and HNPCC group for CiMP-H based on the provisional result from this study. This would require 376 patients in each arm to demonstrate a significant difference (that is, 0.05 with 80% confidence) if it exists. In addition should such a study be performed incorporating BRAF mutation analysis of polyp DNA along with methylation studies could give definitive conclusions with regards to the nature of HPs in HNPCC.

The true risk of developing CRC in association with HPs in either HNPCC or a sporadic setting is still unknown. Until a large prospective series, similar to the veteran affairs is performed identifying patients with HPs or SAs and defining the incidental risk of CRC this question will remain unanswered. 

The control arm of this study contained an unselected 12-month cohort of specimens collected from the pathology archives at Manchester Royal Infirmary. There are previously no reports of the incidence of methylation in HPs from such a cohort. One of the problems with the current data in the literature is its tendency to report on either patients with hyperplastic polyposis or selected HPs of a certain size or location. In this series, 70% of sporadic HPs demonstrated some degree of methylation (CIMP+); 38% were CIMP-H. This not only supports the current evidence that methylation is a contributing biological mechanism in HPs formation but also demonstrates its high frequency. 

All those MSI-L HPs demonstrated MLH-1 promoter methylation. This supports the hypothesis that HPs rather than adenomas may be the precursor to those MSI+ve sporadic CRCs in the right side of the colon. The association of MSI-L with partial methylation of the MLH-1 promoter has been reported before [28] and may, in a graded fashion, represent a rate limiting step in progression to CRC or contribute to malignant progression [29]. 

Two papers have reported an association of CIMP and MLH-1 methylation in patients with CRC and a family history of CRC [30, 31]. Intuitively it is appealing to consider methylation as a factor increasing an individual's risk of CRC via the serrated pathway in the context of familial CRC due to the association of environmental factors and acquired epigenetic changes. However, in this series less methylation was encountered in the familial group.

## 5. Conclusions

This study has shown that in an HNPCC screening program, HPs that occur in those patients with confirmed mutations or HNPCC on clinical grounds have no more worrying features at a molecular level than those patients with HPs in a sporadic setting. This study has also confirmed that methylation is a common biological event in sporadic HPs. There is a need for longitudinal studies and biological profiling of HPs to clarify the true risk of these lesions progressing to CRC.

## Figures and Tables

**Table 1:** Primers used in the amplification of CpG islands from bisulfite-treated DNA by PCR (nested PCR) including their sequences and annealing temperature.

Gene	Genbank accession number	Sense primer	Antisense primer	Annealing temp (°C)
P16^INK4a^	AF527803 NM_058195	GGTTTTTTTTAGAGG ATTTGAGGGATA	AAACAAACCCTC TACCCACCTAA	62
MGMT	AL355531	GGGTAATTTGGGAG GTAT	CTCTCTTACTTTT CTCAAATCCT	58
MLH-1	U83845	TTAGATTATTTTAGT AGAGGTATATAAG	ATACCTTCAACC AATCACCTCAATA	53

**Table 2:** Primers used in the analysis of methylation at the amplified CpG islands of bisulfite treated DNA by PCR including their sequences and annealing temperature.

Gene	MSP reaction	Sense primer	Antisense primer	Annealing temp (°C)
P16^INK4a^	Unmethylated	TTATTAGAGGG TGGGGTGGATTGT	CAACCCCAAACC ACAACCATAA	64
	Methylated	TTATTAGAGGGTGG GGCGGATCGC	GACCCCCGAACC GCGACCGTAA	77.5
MGMT	Unmethylated	TTTGTGTTTTGATGT TTGTAGGTTTTTGT	AACTCCACACTCT TCCAAAAACAAAACA	65
	Methylated	TTTCGACGTTCGTAG GTTTTCGC	TTTCGACGTTCGT AGGTTTTCGC	70
MLH-1	Unmethylated	TTAATAGGAAGAGT GGATAGTG	TCTATAAATTACT AAATCTCTTCA	57
	Methylated	TTAATAGGAAGAGC GATAGC	CTATAAATTACTA AATCTCTTCG	60.5

**Table 3:** PCR conditions for the amplification step.

Step	Temperature	Duration
Initial denaturation	95°C	4 min
Denaturation	95°C	1 min × 35
Annealing	(Primer specific) °C	1 min × 35
Synthesis	72°C	1 min × 35
Final extension	72°C	7 min
Holding	4°C	—

**Table 4:** PCR conditions for the methylated/unmethylated step.

Step	Temperature	Duration
Initial denaturation	95°C	4 min
Denaturation	95°C	30 sec × 35
Annealing	(Primer specific)°C	30 sec × 35
Synthesis	72°C	1 min × 35
Final extension	72°C	30 min
Holding	4°C	—

**Table 5:** Pyrosequencing primers for MINT 1 and 31.

Gene	Forward	Reverse	Sequencing	Dispensation
MINT 1AF135501	AGGGTTGGAG AGTAGGGGAGTT	ATCTCCCCTCC CCTAATTAACA	GCTTGAGGTTT TTTGTT	CGCTCGTCTCGTATTACTCGT ATACT
MINT 31 AF135531T	TTGGGGTGGG AATTGAGA	CCTCACTTA TTACAAATCC CCACT	GGAATTGAGAT GATTTTAATTT	ATCTAGCTAGCTAGCTCGA

**Table 6:** IHC results of hyperplastic polyps for the mismatch repair proteins.

Staining sporadic/familial	MLH-1	MSH-2	MSH-6
Present	97/52	101/49	77/43
Weak	17/0	14/3	38/9
Patchy	1/0	0/0	0/0

**Total**	**115/52**	**115/52**	**115/52**

**Table 7:** IHC of hyperplastic polyps.

Crypt staining sporadic/familial	Ki 67	CK20
Basal 1/3	50/21	39/18
Middle 1/3	56/28	50/26
Outer 1/3	9/3	26/8

**Total**	**115/52**	**115/52**
